# Usability and Acceptability of an App (SELFBACK) to Support Self-Management of Low Back Pain: Mixed Methods Study

**DOI:** 10.2196/18729

**Published:** 2020-09-09

**Authors:** Anne Lovise Nordstoga, Kerstin Bach, Sadiq Sani, Nirmalie Wiratunga, Paul Jarle Mork, Morten Villumsen, Kay Cooper

**Affiliations:** 1 Department of Public Health and Nursing Norwegian University of Science and Technology Trondheim Norway; 2 Department of Computer Science Norwegian University of Science and Technology Trondheim Norway; 3 School of Computing Science and Digital Media Robert Gordon University Aberdeen United Kingdom; 4 School of Health Sciences Robert Gordon University Aberdeen United Kingdom

**Keywords:** low back pain, self-management, physical activity, exercise, patient education, smartphone, mHealth, eHealth, digital health, case-based reasoning

## Abstract

**Background:**

Self-management is the key recommendation for managing nonspecific low back pain (LBP). However, there are well-documented barriers to self-management; therefore, methods of facilitating adherence are required. Smartphone apps are increasingly being used to support self-management of long-term conditions such as LBP.

**Objective:**

The aim of this study was to assess the usability and acceptability of the SELFBACK smartphone app, designed to support and facilitate self-management of non-specific LBP. The app provides weekly self-management plans, comprising physical activity, strength and flexibility exercises, and patient education. The plans are tailored to the patient’s characteristics and symptom progress by using case-based reasoning methodology.

**Methods:**

The study was carried out in 2 stages using a mixed-methods approach. All participants undertook surveys, and semistructured telephone interviews were conducted with a subgroup of participants. Stage 1 assessed an app version with only the physical activity component and a web questionnaire that collects information necessary for tailoring the self-management plans. The physical activity component included monitoring of steps recorded by a wristband, goal setting, and a scheme for sending personalized, timely, and motivational notifications to the user’s smartphone. Findings from Stage 1 were used to refine the app and inform further development. Stage 2 investigated an app version that incorporated 3 self-management components (physical activity, exercises, and education). A total of 16 participants (age range 23-71 years) with ongoing or chronic nonspecific LBP were included in Stage 1, and 11 participants (age range 32-56 years) were included in Stage 2.

**Results:**

In Stage 1, 15 of 16 participants reported that the baseline questionnaire was easy to answer, and 84% (13/16) found the completion time to be acceptable. Overall, participants were positive about the usability of the physical activity component but only 31% (5/16) found the app functions to be well integrated. Of the participants, 90% (14/16) were satisfied with the notifications, and they were perceived as being personalized (12/16, 80%). In Stage 2, all participants reported that the web questionnaire was easy to answer and the completion time acceptable. The physical activity and exercise components were rated useful by 80% (8/10), while 60% (6/10) rated the educational component useful. Overall, participants were satisfied with the usability of the app; however, only 50% (5/10) found the functions to be well integrated, and 20% (2/10) found them to be inconsistent. Overall, 80% (8/10) of participants reported it to be useful for self-management. The interviews largely reinforced the survey findings in both stages.

**Conclusions:**

This study has demonstrated that participants considered the SELFBACK app to be acceptable and usable and that they thought it would be useful for supporting self-management of LBP. However, we identified some limitations and suggestions useful to guide further development of the SELFBACK app and other mobile health interventions.

## Introduction

Low back pain (LBP) is a common and costly condition, peaking in midlife but affecting all age groups [1,2]. Further, LBP is the leading cause of years lived with disability worldwide [3] and is associated with substantial direct (health care) and indirect (eg, work absenteeism, loss of productivity, disability pensions) costs [4-6]. Most LBP does not have a known pathoanatomical cause and is known as nonspecific LBP [3,7,8]. Clinical guidelines encourage active treatment and self-management [9], with physical activity, patient education, and strength and flexibility exercises being some of the key components [10-12].

Adherence to self-management of LBP is challenging, with several reported barriers, in keeping with other chronic conditions [13,14]. Evidence suggests that individually tailored exercise programs for patients with nonspecific LBP are more effective on pain and functioning than nontailored programs [15]. Mobile health (mHealth) technologies such as mobile apps are increasing in popularity and offer the potential for supporting people with LBP and providing them with individually tailored (personalized) recommendations. Although many mobile apps for self-management of LBP are available, most are not personalized and provide the user with generic recommendations only, which may not be relevant to everyone using the app. Moreover, their effectiveness on pain and functional outcomes has not commonly been documented [16,17].

The selfBACK project aims to improve self-management of nonspecific LBP by developing an evidence-based decision support system (DSS) that is made available for end users via the selfBACK app. The protocol for designing and implementing the selfBACK DSS has been published elsewhere [18]. In brief, the selfBACK app is underpinned by behavior change [19] and normalization process theories [20] and aims to achieve improved clinical outcomes for people with nonspecific LBP of any duration. It does this by promoting behavioral change to address factors such as fear avoidance and low pain self-efficacy. The selfBACK app provides the patient with weekly self-management plans, tailored to their individual characteristics, achieved by gathering data on physical activity (measured by a wrist-worn monitor) and other personal and LBP-related factors (measured by a weekly questionnaire in the app) such as pain, function, sleep, mood, fear avoidance, self-efficacy, barriers, and exercise adherence in the last week [21]. Creating and tailoring of the self-management plans are achieved by using a case-based reasoning methodology to capture and reuse information from previous similar and successful LBP patients (ie, favorable progression of symptoms, measured via the weekly questionnaire) available in the selfBACK case base [21]. In keeping with recent evidence-based guidelines for LBP [10], the weekly self-management plans comprise physical activity recommendations, strength and flexibility exercises, and patient education. Patients can adjust recommended physical activity goals, and exercise prescription is by co-decision, where the app recommends a plan which the patient can adjust if they wish. As there is insufficient evidence on the effectiveness of a specific type of exercise for treatment and prevention of LBP [22], selfBACK incorporates a suite of flexibility and strengthening exercises commonly utilized in the management of LBP. The patient education is also tailored to the individual, depending on their response to the weekly questionnaire. Several behavior change techniques [23] are incorporated in the app, including goal setting, problem solving, feedback and monitoring, commitment, information about health consequences, and prompts and cues.

Following the Medical Research Council guidance for the development and evaluation of complex interventions [24], we took a systematic approach to developing the selfBACK DSS, involving usability and acceptability testing prior to piloting a randomized controlled trial (RCT) [25]. In this paper, we present the results of the usability and acceptability study. Due to the complexity of the selfBACK DSS and the iterative design process employed in its development, we employed a 2-stage approach. In the first stage, we explored the feasibility and acceptability of data collection of patient characteristics and symptoms using a web questionnaire and usability and acceptability of the physical activity component of selfBACK using a wrist-worn monitor and prototype app, Traxivity [26]. In the second stage, we explored the usability and acceptability of a further developed app version with 3 self-management components (ie, physical activity, exercises, and patient education). This manuscript presents summary findings from Stage 1 to demonstrate the iterative design process. Because Stage 2 used a more developed app with all 3 selfBACK components, we present more detailed analysis of this stage.

## Methods

### Study Design

A sequential exploratory mixed-methods design was applied in this study [27] with participants using the selfBACK app for 4 weeks in both stages. In Stage 1, the following data were collected: completion times for the web questionnaire, user activity from the app, and usability and acceptability (via self-report questionnaire). Semistructured telephone interviews with a subgroup of participants were conducted to explain and interpret quantitative findings. In Stage 2, the following data were collected: usability and acceptability of the app version with 3 self-management components. Semistructured interviews were conducted to further explore usability and acceptability.

### Setting and Participants

Stage 1 took place in Aberdeen, Scotland (November 2017 through February 2018). We recruited 16 adults (aged ≥18 years) with nonspecific LBP of any duration or severity from a university physiotherapy clinic, the university staff and student population by email, and the wider public by media release. Interested participants were referred to the study team and screened for inclusion and exclusion criteria either face-to-face or by telephone by a research assistant trained for the study. Suitable participants attended the university to complete baseline measures and be provided with access to the app. In Stage 1, selfBACK was only available as an Android app, and some participants’ phones would not support the app. In these cases, we loaned smartphones to participants for the 4-week study duration.

Stage 2 took place in Trondheim, Norway (April 2018 to May 2018). We recruited 11 adults (aged ≥18 years) with nonspecific LBP of any duration or severity from a hospital back and neck outpatient clinic and the wider public. Interested participants were referred to the study team and screened for inclusion and exclusion criteria by telephone by a research assistant trained for the study. Suitable participants were asked to complete the baseline web questionnaire before they attended the university to be provided with access to the app. Exclusion criteria for both stages were self-reported serious pathology; terminal illness; serious depression; unable to read, speak, or understand English (the selfBACK app was only available in English); leg pain worse than LBP; unable to perform physical activity or exercises; pregnancy; fibromyalgia; and previous spinal surgery. Because selfBACK is intended for self-management of nonspecific LBP of any duration or severity, the participants in both locations were suitable candidates. All participants provided written, informed consent, and ethical approval was granted by the Robert Gordon University School of Health Sciences (SHS/17/14) and the Regional Committee for Ethics in Medical Research, Mid-Norway (2018/31).

### Questionnaires

Information collected by the web questionnaire forms the basis for creating the self-management plans in selfBACK. The information collected included gender, age, weight and height to calculate BMI, education, employment status, LBP intensity, LBP duration, pain-related disability, activity limitation, pain mannequin, pain self-efficacy, leisure-time physical activity, insomnia symptoms, health-related quality of life, and mental health (Table 1). The web questionnaire was completed before the participants were given access to the app. A web questionnaire was carried out at the end of the 4-week test period, including the 10-item System Usability Scale (SUS) [28] and a 29-item design questionnaire [29]. The SUS has a mix of positive and negative questions scored on a 5-point Likert scale (strongly disagree to strongly agree): We adapted each question to say “selfBACK system” in place of “system” (Textbox 1). The design questionnaire was adapted from that used in a previous study of pain self-management apps [29]. It included items on the design and content of the selfBACK system and incorporated a mix of closed and open questions (Multimedia Appendix 1). In Stage 2, only the participants that volunteered for the interviews completed the SUS and design questionnaire.

**Table 1:** Participant characteristics collected with the web questionnaire.

Characteristics		Stage 1 (n=16)	Stage 2 (n=11)
Age (years), mean (SD, range)		51.1 (13.9, 23-71)	43.0 (7.5, 32-56)
Male/female		10/6	5/6
BMI (kg/m^2^), mean (SD, range)		26.2 (4.2, 18.8-32.8)	25.2 (3.2, 18.8-29.5)
Education ≥13 years, n (%)		7 (47)	10 (91)
**Employment, n (%)**			
	Full-time	7 (44)	10 (91)
	Part-time	4 (25)	0
	Retired	4 (25)	0
	Other	1 (6)	1 (9)
**LBP^a^ intensity past week (0-10), [30]**			
	Average LBP, mean (SD, range)	3.8 (2.0, 1-7)	4.5 (2.3, 1-8)
	Worst LBP, mean (SD, range)	5.3 (2.8, 1-10)	5.7 (2.5, 1-9)
**LBP duration, current episode, n (%)**			
	1 week	1 (6)	0
	4 weeks	3 (19)	1 (9)
	12 weeks	5 (31)	0
	>12 weeks	7 (44)	10 (91)
RMDQ^b^ (0-24), median (range) [31]		5.0 (1-17)	9.0 (1-14)
**Activity limitation, n (%)**			
	Not at work/not at leisure	4 (25)	3 (27)
	Not at work/yes at leisure	4 (25)	3 (27)
	Yes, at work/not at leisure	0	0
	Yes, at work/yes, at leisure	8 (50)	5 (46)
**Leisure-time physical activity [32], n (%)**			
	Sedentary	1 (6)	2 (18)
	Some physical activity	10 (62)	5 (46)
	Regular physical activity	5 (32)	4 (36)
	Regular hard physical activity	0	0
PSFS^c^ (0-10), median (range) [33]		6 (4-9)	4 (1-8)
Number of pain sites (0-9), median (range)		3 (1-5)	2 (1-5)
EQ-5D^d^ (0-100), mean (SD, range) [34]		75.1 (14.1, 40-95)	64.5 (22.7, 20-90)
Insomnia symptoms, n (%) [35]		4 (27)	6 (55)
PSEQ^e^ (0-60), median (range) [36]		49 (14-58)	49 (36-60)
PSS^f^ (0-40), median (range) [37]		12 (3-25)	14.0 (10-23)
PHQ-8^g^ (0-24), median (range) [38]		3 (0-13)	7.0 (0-12)

**^a^**LBP: low back pain.

^b^RMDQ: Roland-Morris Disability Questionnaire.

^c^PSFS: Patient-Specific Functional Scale.

^d^EQ-5D: Euroqol 5-D (health-related quality of life).

^e^PSEQ: Pain Self-Efficacy Questionnaire (2-item).

^f^PSS: Perceived Stress Scale.

^g^PHQ-8: Patient Health Questionnaire.

**System Usability Scale.**

1. I think that I would like to use the selfBACK system frequently.

2. I found the selfBACK system unnecessarily complex.

3. I thought the selfBACK system was easy to use.

4. I think that I would need the support of a technical person to be able to use the selfBACK system.

5. I found the various functions in the selfBACK system were well integrated.

6. I thought there was too much inconsistency in the selfBACK system.

7. I would imagine that most people would learn to use the selfBACK system very quickly.

8. I found the selfBACK system very cumbersome to use.

9. I felt very confident using the selfBACK system.

10. I needed to learn a lot of things before I could get going with the selfBACK system.

### Interviews

The semistructured interviews in both stages explored participants’ perceptions of and experiences with using selfBACK and their suggestions for improving it. The interviews covered the following topics: perceived usefulness and appeal of the app; barriers and facilitators to using the app; technical difficulties; app features that were liked or disliked; usability and interactions required from the user; ease of using the wrist-worn physical activity monitor; usefulness of the physical activity, exercise, and educational components; appropriateness of the feedback feature (motivational notifications); general ease of use and navigation; and suggestions for improvement.

In Stage 1, 10 of the 16 participants volunteered for the interviews, which were conducted by 2 research assistants trained by KC. In Stage 2, 10 of the 11 participants volunteered for the interviews, which were conducted by ALN. Stage 2 interviews were conducted in Norwegian and translated to English for analysis.

### Data Analysis

Descriptive statistics are reported for demographics, completion rates for the web questionnaire (Stage 1), app user activity (Stage 1), and the design questionnaire. An overall SUS score was computed for each participant in keeping with previous research [39], such that the final scores ranged from 0 to 100, with higher scores indicating better usability. Interviews were transcribed and then analyzed by ALN and KC using framework analysis [40], which is increasingly used in health research as a systematic tool for thematically analyzing interview data [41]. This involved familiarization with the data (reading and rereading transcripts), coding the data (using both predefined and open coding using Microsoft Word), developing an analytical framework (grouping codes into categories in an iterative manner), applying the analytical framework to the whole data set, charting the data in Microsoft Excel (arranging summaries of the data in matric-based charts according to key themes and subthemes), and finally interpreting the data, within and between participants [41]. At each stage, the researchers worked independently on a sample of data (2 or 3 transcripts) before comparing and reaching agreement and then completing the remaining stage independently, before again discussing and reaching agreement. For example, ALN and KC independently coded 3 transcripts, and they met to discuss coding. Agreement was good (not formally measured); therefore, the remaining transcripts were divided between ALN and KC for coding, with a final meeting to review and make amendments where required.

## Results

Table 1 shows the characteristics of the study samples in Stages 1 and 2, assessed by the web questionnaire. In Stage 1, 16 participants with a mean age of 51.1 years (SD 13.9 years) took part, and 10 participants with a mean age of 43.0 years (SD 7.5 years) took part in Stage 2 (Table 1). There were no dropouts, but there was variability in interaction with the app (see the following sections). In both stages, most participants reported to have LBP for 12 weeks or more during the current episode and low to moderate levels of pain-related disability (ie, median Roland-Morris Disability Questionnaire scores were 5.0 [range 1-17] and 9.0 [range 1-14] in Stages 1 and 2, respectively).

### Stage 1: Web Questionnaire and App Version With the Physical Activity Component

It took on average 18 minutes (range 10-38 minutes) to complete the questionnaire. Participants reported that the completion time was acceptable (14/16, 88%) and that it was easy to complete (15/16, 94%). Just over half (9/16, 56%) found the questions relevant, with 44% (7/16) being unsure.

The average step count goal set by participants was 7004 steps per day (SD 2932, range 3000-12,500 steps per day), with participants achieving on average 5496 steps per day (SD 4354, range 133-20,791 steps per day). The selfBACK app was opened an average of 6.2 times per day (SD 11.8, range 0-95 times per day), with participants receiving an average 1.8 motivational notifications per day (SD 2.4, range 0-10 motivational notifications per day). Over the course of the study, a total of 569 notifications were sent to the 16 participants. Participants opened 42% (239/569) of the notifications they received, with the notifications sent at the start of the day being opened most frequently. Of the opened notifications, 90% (215/239) were liked by participants, 8% (19/239) were disliked, and no sentiment was expressed on only 2% (5/239). Notifications regarding full goal achievement were most frequently liked.

#### System Usability Scale

The mean SUS score was 64.7 points (SD 21.2, range 10-95 points). Inspection of individual SUS scale items identified low ratings for items 5 and 6: functions being well integrated and inconsistency. Key findings from the design questionnaire that informed further development of the selfBACK system were as follows: Approximately one-third of participants experienced technical difficulties with downloading, installing, or using the app or with synchronizing the wrist-worn activity monitor with their smartphone. The step count information was reported as useful by 60% (10/16) of participants, although only 50% (8/16) perceived it to be accurate. There was a lack of agreement on the number and appropriateness of motivational notifications, but the timing was considered appropriate (10/16, 60%), and they were perceived as personalized (13/16, 80%). Several suggestions were made by participants, which informed further development of selfBACK. Finally, 69% (11/16) said they would download the selfBACK app, and 63% (10/16) would recommend it to a friend.

#### Semistructured Telephone Interviews

Of the 16 participants, 10 volunteered to take part in telephone interviews (mean age 51 years, 60% [6/10] male). Findings largely reinforced those from the electronic survey. In addition, several barriers and facilitators to using selfBACK were identified, which included older age, disabilities, older smartphones, and having to carry the smartphone constantly (for participants who had difficulty synchronizing the activity monitor). Facilitators included the motivational notifications, especially when they were contextualized to the individuals; daily reports about physical activity and goal achievement; and selfBACK being recommended by a health professional.

### Stage 2: Web Questionnaire and App Version With 3 Self-Management Components

Completion times for the web questionnaire were not recorded but all participants reported the time taken as acceptable. They also reported the questions as relevant (8/10, 80%) and easy to answer (10/10, 100%). The mean SUS score was 70.5 points (SD 20.5, range 45-95 points), indicating better usability than the prototype used in Stage 1. Scores on the individual SUS items showed that participants found selfBACK easy to use, felt confident using it, and thought most people would learn to use it quickly. Of the 10 participants, 9 agreed that they would “like to use the selfBACK system frequently.” In general, they did not find the app to be cumbersome and did not require the support of a technical person. However, only 50% (5/10) found the functions to be well integrated, and 20% (2/10) found selfBACK to be inconsistent, suggesting that further development was required prior to testing in an RCT.

Responses to the design questionnaire showed that most participants (7/10, 70%) found the overall design and appearance of the app attractive or very attractive, with 20% (2/10) reporting it as unattractive and suggesting a more colorful layout. The professionalism of the layout was found attractive or very attractive by 60% (6/10) of participants. General comments on appearance and design were that the content was well-written and clear, but that the colors could have been more attractive. Furthermore, participants found the exercise and physical activity components most useful, with 80% (8/10) rating them as useful or very useful. The education component was rated as useful or very useful by 60% (6/10) of participants. The information on step count and goals was rated as useful or very useful by 50% (5/10) of participants. There was less agreement on the usefulness of the motivational notifications; only 50% (5/10) found them useful, whereas 30% (3/10) found them not useful. Most participants (6/10, 60%) were neutral on whether the app was helping them to self-manage their LBP, whereas 20% (2/10) found it useful and 20% (2/10) did not find it useful. The weekly tailoring questions asked in the app were only found relevant by 20% (2/10) of participants, 50% (5/10) were neutral, and 30% (3/10) found them irrelevant. However, all participants found the questions easy to answer and the time to complete them was acceptable. General comments on the usefulness and content of the app were that the amount of information in the educational module was appropriate, and the app was easy to use. Overall, the app was considered useful; however, there were too many technical challenges. Some suggestions were made by the participants to improve the app, such as making the app more attractive by using more colors and to include the ability to go back in time to review statistics and previous self-management plans.

#### Semistructured Telephone Interviews

Four themes emerged during analysis of the interview data: (1) Practical and Technical Factors, (2) Limitations and Barriers, (3) Strengths and Facilitators, and (4) Suggestions for Improvement. Each theme is presented in the following paragraphs.

The practical and technical factors were related to wearing the physical activity monitor and general difficulties using the app. No issues were reported with charging the physical activity monitor and most participants wore the wristband either all the time, or they only took it off at night:

…as this was soft, it was ok to use. I am thinking about buying something like that…it was a bit uncomfortable at night, as I am not used to wearing anything.

Participant 03, Female, 37 years

Yes, I have [worn it all the time]. It worked out well. And it was useful that it showed the time as well, it motivated me to wear it.

Participant 10, Female, 32 years

It was reported to be uncomfortable by one participant, but not to the extent that she stopped using it:

It is a bit uncomfortable, as it is a bit wide and pointy, but it worked out fine.

Participant 08, Female, 37 years

Most participants experienced some technical difficulties, either with initial login and synchronizing with their smartphones or with the app freezing or shutting down unexpectedly during the 4-week period. Most participants continued to use the app, but in two cases, they stopped: one due to persistent log-in difficulties and the other due to not receiving any self-management plan for exercises after week one.

Aside from technical difficulties, limitations and barriers were related to app content, appearance, and LBP symptoms. Participants were generally positive about the appearance of the app, but some felt it could be enhanced:

I don’t think it [layout and design of the app] catches me that well.

Participant 08, Female, 37 years

And the notifications could also be a bit more colorful. I don’t know much of app development, but I believe that to get people addicted, you need a lot of colors and that it looks fancy or something like that. I did sometimes think that maybe it is a bit too boring?

Participant 10, Female, 32 years

Some participants perceived that their step count was inaccurate, compared to other devices they were concurrently using, and one participant was unable to set a step count goal. Most participants were positive about the suggested exercises; however, one felt they were aimed at older people, one felt they were not tailored to her symptoms, and one would have liked more variety. Participants felt the educational material was easy to understand, but for some it was too simplified, and they were ambivalent about its usefulness:

I think it was a bit trivial information. But it is good to have it in the app. I will not say I learned a lot, but it was OK information to read

Participant 09, Male, 37 years

Most participants found the notifications to be a limitation of the app. They either received very few, they perceived them as irrelevant because of unsynchronized step count, or they found them not to be motivational. However, they could generally see their benefit:

Yes, if synchronized well [would want more notifications], maybe a couple of times each day would be appropriate.

Participant 07, Male, 56 years

One participant felt his LBP symptoms prevented him from using the app as much as desired:

…the usage decreased due to some sleep problems. So, I don’t feel I have been using it optimally. I have tried some days where I only used the stretching exercises and reduced the walking. This was somewhat better, but as soon as I started with the walking again, especially on undulant terrain, I had to lie down again for a couple of days afterwards.

Participant 01, Male, 44 years

Strengths and facilitators were related to content and appearance. Participants commonly reported that they liked the simple and easy-to-understand design. They also liked the visual representation of progress towards goal achievement:

I think it was nice with the “pies” [pie chart]. I have to fill the pie before I go to sleep. That was very good and motivational and fun.

Participant 03, Female, 37 years

Most participants reported the exercises as a strength of the app. They found them to be relevant and liked the instructional videos:

[The videos] was a very good thing. You do wonder if you are doing the exercises correct. The videos were clear and not too fast.

Participant 06, Male, 43 years

Despite the perceived limitations with the step count function reported in an earlier paragraph, some participants particularly valued this function:

I have used the step count a lot, usually on a daily basis. I tried to complete the daily step goal. It was easy to use and an apparent way to see how active you are. It was a barrier to perform the back exercises often enough, but I felt it [step goal] was OK, and it could easily be combined with other things during the day.

Participant 09, Male, 37 years

Not all participants received trophies (for achievements) due to technical issues; however, those who did receive them reported them to be motivating.

A number of suggestions for improvements were made by participants. Two participants felt that sleep monitoring would be a useful addition, and several participants wanted to view their history:

The only thing I could have wanted was the ability to go back in time to look at history of activity level. Because that is a good motivator.

Participant 02, Female, 52 years

More varied content was generally wanted by participants, including more variety of exercises, the ability to select or deselect exercises, and information on calories burned during physical activity. Finally, more colors and more “fancy” layout was suggested by one participant.

## Discussion

### Principal Findings

This 2-stage usability and acceptability study generated important knowledge for the further development of the selfBACK app for use in a pilot study and full RCT [25]. Stage 1 demonstrated acceptability of the web questionnaire; however, due to relatively low numbers of participants reporting the questions as relevant, review of participant information to accompany the web questionnaire was warranted. Interaction with the app was variable, and usability could be enhanced, with several participant-identified limitations and suggestions useful for enhancing the next version of the app. Nonetheless, we were encouraged by the high levels of participants who would download the selfBACK app and recommend it to a friend.

Stage 2 demonstrated enhanced usability and identified areas where further development was required, particularly educational content, perceived step count accuracy and goal setting, motivational notifications, and overcoming technical barriers to using the app. Overall, we demonstrated that the selfBACK app was usable and acceptable to people with LBP and with further development, was suitable for piloting in a clinical population.

There were some conflicting results regarding the motivational notifications; most participants in Stage 1 liked the notifications they opened, whereas only 50% (5/10) of participants in Stage 2 found them to be useful. The notifications were developed at the app design stage by consulting with potential app users to gather suggestions of what they would find motivational. We found it challenging to make final selections, as feedback from our user groups reinforced that liking or not liking notifications is highly subjective and personal. For instance, some participants in Stage 1 found motivational messages to be beneficial, specifically as they were often linked to the participant’s own step count, while others found that timing of messages was not meaningful and failed to grab attention. There was also general consensus that further information such as educational content on LBP was needed. Notifications have been found to enhance engagement with health apps [42], with tailored suggestions most effective [43]. In summary, these studies highlighted the need for messaging logic to be contextually relevant to the participant both in terms of content and timelines. Thus, notifications for the selfBACK app were further refined following the recommendations in these studies, and the possibility to turn on and off notifications was added.

Participant-identified limitations have been used in the continued development of selfBACK, such as adding more content with a wider selection of educational material and exercises, adding an option to look back at one’s own data to see history of activity level, adding an option for skipping or replacing exercises at the participant’s preference, and changing how the step goals were created by taking the last 2 weeks into account instead of only the last week. The technical barriers experienced by participants also enabled the study team to make improvements, both to the app and accompanying user instructions, in keeping with the iterative design process adopted. Despite technical and other barriers reported by participants, overall usability (mean SUS score 70.5 in Stage 2) was good [44]. We are confident that further development of the selfBACK app, in line with the findings of this study, will enhance overall usability.

### Limitations

The sample sizes in both stages were small and were comprised of participants with mild to moderate chronic LBP with the ability to read and understand English as the app was only available in English at this stage. selfBACK is intended for use in 3 languages (English, Danish, Norwegian) and for people with a range of LBP severity and duration. However, it is not the intention to generalize from usability and acceptability studies, and selfBACK is being further tested in a pilot study and a full RCT [25], in keeping with guidance for the development of complex interventions [45].

### Conclusions

We have demonstrated acceptability and usability of selfBACK, an evidence-based DSS for supported self-management of LBP delivered by a smartphone app, in a sample of people with non-specific LBP. Technical reliability, ability for individual adjustments, relevant educational content, and targeted notifications were highlighted as important for enhancing usability of the selfBACK app. Based on these results, a further refined version of selfBACK could be piloted [46] in order to determine whether a full-scale RCT should be conducted. Future research should focus on appropriate and effective tailoring of mHealth notifications to individual’s needs and preferences, in order to develop truly personalized interventions.

## Appendix

Multimedia Appendix 1 The design questionnaire used in the present study.

## Abbreviations

DSS: decision support system
EQ-5D: Euroqol 5-D (health-related quality of life)
LBP: low back pain
PHQ-8: Patient Health Questionnaire
PSEQ: Pain Self-Efficacy Questionnaire
PSFS: Patient-Specific Functional Scale
PSS: Perceived Stress Scale
RCT: randomized controlled trial
RMDQ: Roland-Morris Disability Questionnaire
SUS: System Usability Scale
