# Supplementary material for: Usability and Acceptability of an App (SELFBACK) to Support Self-Management of Low Back Pain: Mixed Methods Study
Source: JMIR Rehabil Assist Technol. 2020 Sep 9;7(2):e18729. doi: 10.2196/18729 (PMC7511856; doi:10.2196/18729)
Supplement: Multimedia Appendix 1 [file rehab_v7i2e18729_app1.docx]

Adapted from Reynoldson et al (2014) Assessing the Quality and usability of Smartphone Apps for Pain Self-Management, Pain Medicine, DOI: 10.1111/pme.12327

**Look**

**Each item uses a five-point Likert response format with wording on the poles (e.g., for item 12 “very unattractive” scores 1 and “very attractive” scores 5).**

How attractive did you find the use of colours in the app?

How attractive did you find the fonts used in the app?

How pleasant did you find the layout of the app?

How professional did you feel the app was to look at?

To what extent do you feel the design suited the purpose of the app?

How pleasant did you find the look of the app overall?

Overall, how much did you like the design of the app?

Do you have any overall comments on the design of this app?

**Content**

**Each item uses a five-point Likert response format with wording on the poles (e.g., “not useful at all” scores 1 and “very useful” scores 5).**

How useful did you find the exercise section

How useful did you find the education section?

How useful did you find the physical activity part of the intervention?

How useful was the information you received about step count/physical activity?

How useful was the information about goal achievement?

How useful were the physical activity reminders?

How useful were the motivational messages?

How sufficient did you feel the content of the app was in enabling you to self-manage your low back pain?

Do you have any overall comments about the content of this app?

**Questionnaires**

How relevant did you find the baseline questionnaires for describing your back problems (those you completed before using the app for the first time)? (5-point Likert Response)

Were the baseline questionnaires easy to complete? (Yes/No)

Was the time taken to complete the baseline questionnaires acceptable? (Yes/No)

How relevant did you find the follow-up questionnaires for describing your back problems (those you completed each week)? (5-point Likert scale)

Were the follow-up questionnaires easy to complete? (Yes/No)

Was the time taken to complete the follow-up questionnaires acceptable? (Yes/No)

Do you have any overall comments on the questionnaires?

**Suggestions**

Score “yes” or “no” with free text comments below.

Is there anything you would add to this app to make it more useful for self-management of low back pain?

Is there anything in this app you felt was unnecessary or could be removed?

Is there anything you particularly liked about this app?

Is there anything you particularly disliked about this app?

Do you have any other suggestions?

**Future use**

Score “yes” or “no”

Would you download this app?

Would you use it long-term (more than 3 months)?

Would you recommend this app to a friend?

Do you have any final comments about this app?
